# What Predicts Korean Citizens’ Mask-Wearing Behaviors? Health Beliefs and Protective Behaviors against Particulate Matter

**DOI:** 10.3390/ijerph18062791

**Published:** 2021-03-10

**Authors:** Jarim Kim, Yerim Kim

**Affiliations:** 1 Department of Communication, Yonsei University, Seoul 03722, Korea; jarimkim@yonsei.ac.kr; 2 Department of Neurology, Kangdong Sacred Heart Hospital, College of Medicine, Hallym University, Seoul 05355, Korea

**Keywords:** particulate matter, health belief model, perceived susceptibility, perceived severity, perceived benefits, perceived barriers, mask-wearing, health promotion, health communication

## Abstract

Air pollution has become a critically important contemporary issue, exposing people to various health risks worldwide. Air pollution problems cannot be resolved in the short-term; therefore, citizens in regions with low air quality are encouraged to take protective actions such as wearing masks to filter particulate matter. However, compliance with such recommendations is limited. To enhance the effectiveness of health promotion in this regard, this study applied the health belief model to examine the factors that affect mask-wearing behaviors. It also investigates the factors that influence particulate matter-related health beliefs and protective behaviors. A cross-sectional survey with 200 Korean citizens was conducted. The results revealed associations between masking behaviors and both perceived benefits of and perceived physical barriers to wearing masks. In addition, sex, education, income, and having heard of different particulate matter-related health consequences were found to predict mask-wearing. This study demonstrates the utility of the health belief model in the context of air pollution and has practical implications for health promotion practitioners.

## 1. Introduction

Every year, more than four million people worldwide lose their lives prematurely due to heart disease, pneumonia, stroke, chronic obstructive pulmonary disease, and lung cancer caused by outdoor air pollution [1]. Approximately 92% of people worldwide live in environments where air quality levels do not meet standards set by the World Health Organization (WHO). The WHO set air quality guidelines as an annual mean of fine particulate matter of (PM) 2.5 of less than 10 μg/m^3^ [2]. Particle matter (PM), comprising small solid or liquid aerosols, is harmful when inhaled [3]. PM_2.5_, fine, inhalable particles that are 2.5 micrometers or smaller in diameter [4], are dangerous because they can penetrate more deeply into the body and reach the blood or lungs. PM exposure is linked to various health effects, ranging from eye irritation and breathing trouble to heart disease and lung cancer [5]. One study ranked PM_2.5_ fifth among 79 risk factors responsible for global diseases in 195 countries [6].

PM issues have emerged as a major concern in Korea. According to 2019 data concerning population exposure to PM_2.5_, Korea ranked highest among the Organization for Economic Cooperation and Development (OECD) countries at 27.5 μg/m^3^, almost doubling the average exposure of other OECD countries (13.9 μg/m^3^) and three-fold higher than the WHO standard (10 μg/m^3^) [7]. Recognizing the risk of such exposure posed to citizens, the Korean government enacted the Special Act on the Reduction and Management of Fine Dust on 14 August 2018 [8], implementing protective measures including notifying citizens of PM-related problems or the PM status in given locations. For example, in 2019, citizens in Seoul, the capital of Korea, received 41 “bad” day (36–75 μg/m^3^) and 9 “very bad” day (more than 76 μg/m^3^) warnings [9]. As protective actions, the government recommends that citizens refrain from outdoor activities and to wear masks certified by the Korean Centers for Disease Control and Prevention, such as N95, KF94, or KF80 types, when then need to go outside. However, for various reasons, including the discomfort or stifling feeling caused by wearing a mask or monetary cost for mask acquisition, some citizens do not comply with governmental guidelines [10], or use other types of masks that may not effectively filter out PM_2.5_ [11].

Considering that this air pollution issue cannot be resolved in a day or a year, it is important to theoretically understand why citizens do not comply with governmental recommendations and identify practical strategies to help them engage in protective actions to reduce the health risks they face. Most PM-related studies have taken more natural scientific or engineering approaches, focusing, for example, on mask-wearing’s efficacy in reducing health risks [12,13], or the links between PM and different diseases [13,14]. Meanwhile, researchers have paid relatively little attention to the variables that influence individuals’ protective behaviors—an area that urgently requires research in the short-term to reduce negative heath consequences. Thus, guided by the health belief model [15] (acknowledged as one of the most useful frameworks for explaining health behaviors [16]), this study examines the factors that affect mask-wearing behaviors.

## 2. Theoretical Framework

### 2.1. Health Belief Model

The health belief model (HBM) was developed in the 1950s to understand why U.S. citizens did not participate in disease prevention programs. It has guided various health behavior interventions by providing a framework for investigating the various health beliefs associated with different health-related issues [17,18], and a considerable number of empirical studies have supported its theoretical and practical utility [19]. Based on the value-expectancy approach, which predicts human behaviors based on one’s evaluation of an outcome and expectation that one’s behavior will result in that outcome [20], the theory postulated that individuals value avoiding diseases and expect that their specific health behaviors will prevent diseases [21].

The HBM comprises the following four basic constructs: (i) perceived susceptibility, which refers to the perceived likelihood of a risk’s occurrence; (ii) perceived severity, which refers to the perceived seriousness of a risk’s consequences; (iii) perceived benefits, which refers to the perceived efficacy of a behavior in reducing a risk; and (iv) perceived barriers, which refers to the perceived psychological, physical, financial, and other costs of engaging in a particular behavior [20,21]. Higher levels of perceived susceptibility and perceived severity have been shown to increase the perceived threat posed by a given risk, whereas higher levels of perceived benefits and lower levels of perceived barriers increase the likelihood of engaging in suggested health behaviors [22]. The theory has been applied to various health contexts, including breast cancer screening [23], condom use [24], H1N1 vaccination [25], HPV vaccination [26], exercising [27], energy drink consumption [28], and smoking [29].

Earlier review studies [30,31] have identified perceived barriers as the strongest predictor and perceived severity as the weakest predictor across different health behaviors. Janz and Becker also found that the associations between health beliefs and behaviors differed depending on the type of health behaviors. Specifically, they found that perceived susceptibility better predicts preventative behaviors, whereas perceived severity and benefits better predict treatment behaviors of already diagnosed diseases. Perceived barriers did not differ across types. However, a more recent meta-analysis focusing only on longitudinal studies [32] identified perceived benefits and barriers as the strongest predictors and perceived severity as a weak predictor. This same study found that perceived susceptibility had almost null associations with health behaviors. In short, findings regarding the strength of the associations between different health beliefs and health behaviors have been inconsistent, and these associations therefore require further research [32,33]. Based on this theoretical framework, the current study examined the effects of health beliefs on protective behaviors against PM—mask-wearing—and the potential antecedents that may affect health beliefs about mask-wearing. Despite its utility in various health contexts, the HBM has rarely, if ever, been utilized to examine individuals’ protective behaviors against air pollution.

This study postulates that mask-wearing behaviors are associated with health beliefs, including the perceived susceptibility to PM-related diseases, perceived severity of PM-related health consequences, perceived benefits of wearing masks in reducing PM-related risks, and perceived barriers to mask-wearing. Most prior studies have regarded perceived barriers as a unidimensional factor, agglomerating different types of barriers including physical, psychological, financial, and other costs [25,34,35]. More recent studies [26,28,36,37], however, have emphasized the multidimensional nature of this concept. Following prior research regarding protective behaviors [26,37], this study distinguishes between the physical and financial/logistical barriers to wearing masks. Although no prior studies have directly examined the relationships between PM-related health beliefs and protective behaviors, based on the extant HBM research, this study posits the following hypotheses (Hs):

**Hypothesis** **1** **(H1).** *Perceived susceptibility to PM-related diseases positively predicts mask-wearing behaviors.*

**Hypothesis** **2** **(H2).** *Perceived severity of PM-related health consequences positively predicts mask-wearing behaviors.*

**Hypothesis** **3** **(H3).** *Perceived benefits of wearing masks positively predict mask-wearing behaviors.*

**Hypothesis** **4** **(H4).** *Perceived physical barriers to wearing masks negatively predict mask-wearing behaviors.*

**Hypothesis** **5** **(H5).** *Perceived financial/logistical barriers in wearing masks negatively predict mask-wearing behaviors.*

### 2.2. Predictors of PM-Related Health Beleifs and Protective Behavios against PM

Moreover, this study takes into consideration the diverse sociodemographic characteristics that may affect individuals’ health beliefs and behaviors. The original HBM assumed such relationships [31], but prior studies have neglected them. Although basic, engaging in effective communication requires an understanding of how different segments of the population grouped by sociodemographic characteristics demonstrate different health beliefs and behaviors. For example, younger people may perceive themselves as less likely to develop PM-related disease than older people, or women may find wearing masks less appealing because of how masks look on their faces. Such associations, if supported, mean that different communication messages need to be designed depending on group characteristics. Furthermore, clarifying such relationships will help determine which approaches are necessary to encourage citizens’ mask-wearing. For example, if low-income predicts noncompliance with mask-wearing, policy- or community-level solutions to help low-income individuals acquire and wear masks may be more appropriate than persuading individuals to wear masks.

In line with this, the current study also explores how having heard of PM’s diverse harmful consequences influences PM-related health beliefs and mask-wearing behaviors. People usually associate PM with certain respiratory diseases such as coughs or sore throats, not acknowledging its links to various serious ailments such as cardiovascular diseases or lung cancer. It is possible that simply having heard of or having communicated with others about different health consequences may affect individuals’ health beliefs and resulting behaviors. Thus, the following research questions (RQs) are asked:

**RQ1.**  *Will any of the sociodemographic characteristics and having heard of PM’s harmful health effects impact PM-related health beliefs?*

**RQ2.**  *Will any of the sociodemographic characteristics and having heard of PM’s harmful health effects impact mask-wearing behaviors?*

## 3. Materials and Methods

### 3.1. Subjects and Procedures

A cross-sectional online survey was conducted with Korean adults between January 30, 2020, and February 3, 2020. Previous HBM research [32,33] has shown small to medium effects. A G*Power analysis [38] indicated that a total sample size of 138 would provide 95% power to detect a medium-sized effect (*f* = 0.15) in a multiple regression. Thus, we aimed to recruit at least 150 participants to achieve the requisite power. Subjects were recruited from a survey company EMBRAIN panel that consisted of 1.2 million Koreans. Based on stratified random sampling that represented the characteristics of Koreans in terms of age, sex, and region, a study invitation was sent to 4125 people; 282 completed the survey, and 82 were excluded due to sloppy responses and insufficient participation time, resulting in a final sample size of 200. It took subjects an average of 19.3 min to complete the survey and they were rewarded with EMBRAIN panel points, which can be exchanged for gifts or cash. The study received institutional review board approval before administering the survey and the researchers complied with ethical standards throughout the research.

### 3.2. Measurements

HBM measures were adapted from prior studies [26,37]. Three items were used to measure each of the HBM constructs including perceived susceptibility to PM-related diseases (e.g., “It is likely that I will get cancer or respiratory or cardiovascular diseases due to PM”), perceived severity of PM-related health consequences (e.g., “I believe that the PM will cause severe health problems”), and perceived benefits of wearing masks (e.g., “I believe wearing masks is effective in preventing cancer or the respiratory or cardiovascular diseases caused by PM”). Perceived barriers to wearing masks were measured with two constructs adapted from prior research [26,37], including perceived physical barriers (e.g., “Wearing masks makes it hard for me to breathe”) and perceived financial/logistical barriers (e.g., “I cannot afford masks”, “I do not wear masks because of how silly they look”). Subjects were asked to respond on a 7-point Likert scale ranging from 1 (strongly disagree) to 7 (strongly agree), which were averaged to form an index for each construct.

To measure mask-wearing behaviors, subjects were asked “How many days did you wear masks in the past 30 days?” and “How many days did you wear masks in the past 3 months?” Air quality indexes for the past three months were presented with these questions as follows: “In the past three months, the fine dust index indicates 15 bad days, 60 average days, and 16 good days, while the ultra-fine dust index indicates 18 bad days, 53 average days, and 21 good days.”

To measure the extent to which subjects had heard of PM-related negative health consequences, five questions concerning various health consequences were asked. Sample questions included: “Have you heard that the harmful health effects of PM can include lung cancer?” and “Have you heard that the harmful health effects of PM can include cardiovascular disease that can cause heart attack or stroke?” Subjects responded either 0 (no) or 1 (yes) to each question and the scores were added to form an index. In addition, sociodemographic information including age, sex, having children, education level, and annual income was collected.

### 3.3. Statistical Analysis

To answer the research questions and test the hypotheses, a series of multiple regression analyses were conducted using SPSS Statistics 26 software (IBM Inc., Chicago, IL, USA). To control for the effects of sociodemographic characteristics or having heard of PM’s harmful effects in examining the relationships between mask-wearing and health beliefs, data for the RQs were analyzed before those for the Hs. The severity of air pollution might differ based on the specific regions in which participants resided. Thus, region was controlled throughout the analyses. Specifically, for RQ1, each of the five health beliefs were included as dependent variables and sociodemographic characteristics (i.e., age, sex, having children, education, and income) and the extent to which individuals had heard of PM-related negative health effects were the independent variables. As with the RQ1 analyses, for RQ2, a series of regression analyses were performed with mask-wearing behaviors as the dependent variables and sociodemographic characteristics and the extent to which individuals had heard of PM-related negative health effects as the independent variables.

For H1, five health beliefs were included as independent variables, and mask-wearing behaviors in the past 30 days and in the past 3 months were the dependent variables for each analysis. Sex and having heard of PM’s negative health consequences were included as covariates for the H1 test. To validate the dimension of health beliefs, a confirmatory factor analysis (CFA) was conducted using AMOS Graphics (SPSS software).

## 4. Results

### 4.1. Descriptive Statistics and Dimensionality

Table 1 presents descriptive statistics including sociodemographic characteristics. To validate the dimensionality of the five health belief dimensions including perceived susceptibility, perceived severity, perceived benefits, perceived physical barriers, and perceived logistical/financial barriers, a confirmatory factor analysis was conducted. The analysis yielded a very good model fit: *χ*^2^ (81) = 133.78, root mean square error of approximation (RMSEA) = 0.057, comparative fit index (CFI) = 0.976, Tucker–Lewis Index (TLI) = 0.969. An RMSEA smaller than 0.06 and a CFI and a TLI larger than 0.90 and 0.95, respectively, are suggested benchmarks for a good fit.

### 4.2. Main Results

RQ1 asked if any of the sociodemographic characteristics and having heard of PM’s negative health consequences were associated with PM-related health beliefs. As shown in Table 2, the analysis indicated that females were more likely to perceive PM-related consequences as more severe. Education and having heard of negative PM-related health consequences were also shown to have positive relationships with perceived susceptibility to and severity of PR-related consequences. Income, on the other hand, showed a negative relationship with perceived physical barriers to mask-wearing.

RQ2 asked if any of the sociodemographic characteristics and having heard of PM’s harmful health consequences were associated with mask-wearing behaviors. As shown in Table 2, the analysis showed that females were more likely to have worn masks in the past 30 days as well as past 3 months. Having heard of different negative PM-related health consequences was also found to be positively associated with mask-wearing behaviors, both for the past month and the past 3 months.

H1–5 posited that health beliefs predict mask-wearing behaviors. Sex and having heard of negative health consequences caused by PM were related with mask-wearing behaviors; therefore, these two variables were included as covariates during analyses. As shown in Table 3, the perceived benefits of wearing masks were positively associated with mask-wearing for the preceding one and three months, and perceived physical barriers were negatively associated with mask-wearing for the previous one and three months, supporting H3 and H4. Meanwhile, no associations were found between mask-wearing behaviors and perceived susceptibility, perceived severity, and perceived financial/logistical barriers. Thus, H1, H2, and H5 were rejected.

## 5. Discussion

This study examined how people’s health beliefs influence their mask-wearing behaviors to protect themselves from PM and whether sociodemographic characteristics and having heard of PM’s harmful health consequences affect health beliefs and protective behaviors. The analysis showed that perceived benefits of wearing masks in reducing PM-related risks and perceived physical barriers to mask-wearing predict mask-wearing behaviors. It also showed that sex, education, income, and having heard of harmful effects predict health beliefs and mask-wearing behaviors.

The current study demonstrates the utility of HBM in predicting mask-wearing behaviors. Perceived benefits of and perceived physical barriers to wearing masks increased the likelihood of mask-wearing, but perceived susceptibility to PM-related diseases and perceived severity of PM-related health consequences did not, confirming the findings of an HBM meta-analysis [32], but undermining the argument of an earlier review [31] which perceived that susceptibility effectively predicts preventative behaviors. The findings of this study run counter to speculative assertions made in other previous studies as well [31,33]. These meta-analyses expected the predictive power of perceived severity to be weak based on the rationale that most individuals would perceive diseases such as AIDS or breast cancer as extremely severe. They predicted that this lack of variance between individuals would lead to small effect sizes. The variance in perceived severity in the current study, however, was even larger than the variance in other beliefs that showed statistically significant associations with behaviors, disconfirming such speculation.

One potential explanation for the null associations between mask-wearing and perceived severity and susceptibility is the uncertainty surrounding PM-related diseases. PM exposure has been linked to a number of potential health consequences, including cardiovascular and respiratory diseases, rather than a single concrete disease, and these diseases differ in seriousness (e.g., lung cancer vs. cough). Unlike some other health behaviors that prevent certain diseases, such as HPV vaccination preventing 70% of cervical cancer, the severity of PM-related consequences and individuals’ susceptibility to them may not be certain. The fact that the findings of prior studies regarding the associations between health behaviors and perceived susceptibility and severity have not been consistent [31,32,39] highlights the need to examine (un)certainty regarding the risks posed in study contexts as a potential moderator.

On the other hand, this study found that perceived benefits and barriers predict mask-wearing, supporting prior studies [25,26,28,31,32,37] that have emphasized the importance of perceived benefits and barriers in predicting health behaviors. Perceived threats posed by susceptibility and severity may play some role in motivating action, but perceived benefits and barriers are the direct motivators that trigger health behaviors, demonstrating stronger associations across studies.

The findings of this study also revealed the multidimensionality of perceived barriers and their differential effects on health behaviors. Unlike the most extant studies, which have treated perceived barriers as unidimensional [25,34,35], this study made distinctions between physical barriers stemming from wearing masks and other barriers related to monetary costs or the logistics of wearing masks. The findings showed that only physical barriers predicted behaviors, implying that fully understanding health behaviors will require examination of different barriers. The findings also support prior studies that revealed different dimensions of perceived barriers [26,28,37]. The fact that studies have consistently found a close relationship between perceived barriers and health behaviors [32] emphasizes the critical importance of identifying context-specific barriers that need to be removed to promote health behaviors. For example, in the current context, removing physical barriers is important to increasing mask-wearing.

The current findings need to be interpreted in the Korean context. For example, the findings revealed that logistical/financial barriers are not big issues for Koreans. However, at the time, Koreans had experienced serious air pollution issues for several years, been exposed to media coverage highlighting the issue’s seriousness, and became familiar with purchasing and wearing masks; these factors may explain this finding. Thus, logistical/financial barriers may prove more serious in other countries and deter citizens there from engaging in protective behaviors. For example, wearing masks may be viewed as culturally unacceptable in other cultures, or certified masks may cost much more, increasing related barriers. Additional research in this regard is therefore warranted.

Notably, this study found that a number of sociodemographic characteristics predict different health beliefs and mask-wearing behaviors. Specifically, females perceived PM-related diseases as more severe, which made them more likely than males to wear masks. Those with higher levels of education perceived PM-related consequences as more severe and perceived themselves as more susceptible. Research has consistently shown a positive relationship between education and knowledge, because education enhances the skills necessary to learn and integrate necessary information [40,41,42,43]. The fact that PM-related knowledge is more accessible to those with higher education levels explains their higher levels of perceived susceptibility and severity. Interestingly, income was negatively associated with perceived physical barriers. The stuffiness or discomfort caused by wearing masks did not differ based on income levels. A possible rationale for this is that lower income-level jobs involve more outdoor work and therefore the workers in these jobs have to wear masks longer than their higher-earning counterparts. Additionally, such lower income jobs may involve more strenuous physical work, which may cause workers in these jobs to feel breathing difficulties caused by masks more acutely during work.

The current study also found that having heard of PM’s diverse negative health consequences increased the levels of perceived susceptibility, perceived severity, and protective behaviors. Simply having heard about a given disease increased perceived risk levels and affected actual behaviors. This finding highlights the importance of communication. Exposure to simple information via different sources such as doctors, mass media, or social media can make people more aware of the given risks and lead them to engage in protective behaviors.

### 5.1. Practical Implications

The findings of this study have implications for health communication practitioners working in the air pollution context. Firstly, health communication practitioners need to design their key messages to highlight the perceived benefits of wearing masks and reducing perceived physical barriers. Although breathing can be uncomfortable with a mask on, people’s physical discomfort may be based on preconceptions to some extent. In particular, one-fourth of the subjects in this study had not worn masks in the preceding 30 days, and 23% had not during the preceding 3 months. Helping them first experience mask-wearing would be another way to promote protective behaviors. In line with this, masks that make breathing more comfortable are currently being produced. Providing information regarding such options should also increase individuals’ protective behaviors.

Secondly, practitioners need to develop different target-specific messages. For example, males perceived PM as less severe, and they were less likely to wear masks. Thus, mask-wearing promotions should target males and endeavor to increase their perceived severity. Majority-male workplaces or boys’ schools could be good places to practice such promotions.

Thirdly, practitioners should work to inform citizens about PM’s diverse negative health consequences. This study found that education and simply having heard of PM’s negative health consequences were associated with perceived susceptibility and severity, revealing the need for awareness campaigns. This could be an opportunity for practitioners because it is more cost-effective than other communication efforts such as those seeking to change tobacco addiction-related behaviors, where motivating behavioral change is particularly difficult because people tend to maintain such behaviors even though they know about their negative consequences.

### 5.2. Limitations and Future Research Directions

Several limitations warrant mentions. Firstly, this study employed a retrospective design. Although two behavioral questions (i.e., 30 days, 3 months) were asked and participants’ responses were consistent across them, their recollections of the numbers of times they had worn masks may not have been accurate. Future studies should consider different approaches such as having participants keep diaries over three months so as to monitor mask-wearing behaviors using panel data. In line with this, the participants were rewarded for their participation, which may have elicited biases in sample selection or responses (e.g., socially desirable responses). Future studies also need to consider more naturalistic designs that allow researchers to observe individuals’ beliefs and behaviors.

Secondly, this study was conducted in Korea. Perceptions concerning mask-wearing differ across countries and cultures [44]. To enhance the generalizability and practical utility of this study’s findings, researchers should endeavor to replicate it in locations with different cultural backgrounds. In areas with different cultural backgrounds, different health beliefs or sociodemographic characteristics may predict mask-wearing behaviors.

Lastly, the current study reflected citizens’ perceptions and behaviors before the COVID-19 pandemic. COVID-19 has made use of masks a compulsory social norm. How such cultural shifts in mask usage will affect protective behaviors against PM pollution remains unclear. Citizens may have become more familiar with wearing masks and regard it as less bothersome. Alternatively, they may perceive wearing masks to protect against PM as less compulsory and important compared to COVID-19 and therefore enjoy the freedom of non-wearing. Air pollution will still be an issue even after the current pandemic ends, and understanding how and why citizens’ protective behaviors change remains crucial.

## 6. Conclusions

This study investigated how different health beliefs regarding PM affect citizens’ protective behaviors, and what variables influence PM-related beliefs and protective behaviors. This study found that the perceived benefits of and physical barriers to wearing masks predict protective mask-wearing behaviors. Additionally, it revealed that sex, education, income, and having heard of PM’s harmful effects predict PM-related health beliefs as well as mask-wearing behaviors. As the first to examine self-protective mask-wearing behaviors through the HBM lens, the current study has expanded the applicability of HBM and provides practical insights for health promotion practitioners.

## Figures and Tables

**Table 1:** Descriptive statistics.

Variables (*n* = 200)	Levels	Frequency	Percentage
Sex	Male	98	49%
	Female	102	51%
Having children	No	83	41.5%
	Yes	117	58.5%
Education	Less than high school	2	1.0%
	High school diploma	25	12.5%
	Some college	30	15%
	Bachelor’s degree	108	54%
	Postgraduate degree and above	35	17.5%
Annual income	Less than $ 20,000	30	15%
	$20,000–30,000	39	19.5%
	$30,000–40,000	39	19.5%
	$40,000–50,000	30	15%
	$50,000–60,000	22	11%
	$60,000–70,000	13	6.5%
	$70,000–80,000	9	4.5%
	More than USD 80,000	18	9%
	Cronbach’s alpha	***M***	***SD***
Age		44.04	13.27
MW for the past 30 days		8.38	8.36
MW the past 3 months		19.10	20.20
HHH		4.51	0.87
Perceived susceptibility	0.93	4.92	1.23
Perceived severity	0.93	5.60	1.03
Perceived benefits	0.93	5.15	0.94
Perceived physical barriers	0.88	5.53	0.93
Perceived financial/logistical barriers	0.73	2.96	1.16

Note. *M*: mean; *SD*: standard deviation; MW: mask-wearing; and HHH: having heard of harmful health consequences.

**Table 2:** Predictors of PM-related health beliefs and mask-wearing behaviors.

Predicting Variables	Perceived Susceptibility	Perceived Severity	Perceived Benefits	Perceived Physical Barriers	Perceived Logistical/ Financial Barriers	MW for the Past 30 Days	MW for the Past 3 Months
Sex	0.021	0.164 *	−0.028	0.001	−0.018	0.196 **	0.179 *
Age	0.000	0.015	0.000	−0.101	−0.003	−0.150	−0.122
HC	−0.039	0.004	−0.093	0.097	−0.044	−0.037	−0.070
Education	0.180 *	0.176 *	0.023	0.089	0.096	−0.077	−0.040
Income	−0.110	−0.035	−0.009	−0.219 **	−0.009	0.123 ^†^	0.113
HHH	0.193 **	0.245 ***	0.118	0.103	0.043	0.155 *	0.151 *
Region	−0.039	0.040	0.008	−0.089	−0.002	−0.016	−0.003
Total *R*^2^	0.078 *	0.114 ***	0.024	0.060	0.014	0.102 **	0.087 **
Adjusted *R*^2^	0.045 *	0.082 ***	−0.012	0.026	−0.022	0.069 **	0.053 **

Note. Numbers are standardized regression coefficients. HC: having children; MW: mask-wearing; and HHH: having heard of harmful health consequences; Sex: male = 1, female = 2; Having children: no = 0, yes = 1; Education: less than high school = 1, high school diploma = 2, some college = 3, Bachelor’s degree = 4, postgraduate degree or above = 5; Annual Income: below KRW 24 million (i.e., approx. below $20,000) = 1, KRW 24–36 million (i.e., approx. $20,000–30,000) = 2, KRW 36–48 million (i.e., approx. $30,000–40,000) = 3, KRW 48–60 million (i.e., approx. $40,000–50,000) = 4, KRW 60–72 million (i.e., approx. $50,000–60,000) = 5, KRW 72–84 million (i.e., approx. $60,000–70,000) = 6, KRW 84–96 million (i.e., approx. $70,000–80,000) = 7, KRW 96 million and above (i.e., approx. $80,000 and above) = 8; Region: Seoul = 1, Pusan = 2, Daegu = 3, Incheon = 4, Gwangju = 5, Daejeon = 6, Ulsan = 7; Having heard of PM-related negative health effects: higher scores indicate greater levels of having heard of different negative health effects. Significance indicated by ^†^ *p* < 0.10, * *p* < 0.05, ** *p* < 0.01, *** *p* < 0.001.

**Table 3:** Predictors of mask-wearing behaviors.

Predicting Variables	Past 30 Days	Past 3 Months
Sex	0.211 **	0.184 **
Having heard of negative effects	0.131 ^†^	0.107
Region	−0.042	−0.031
Perceived susceptibility	0.134	0.138
Perceived severity	−0.035	0.009
Perceived benefits	0.196 **	0.238 ***
Perceived physical barriers	−0.256 ***	−0.192 **
Perceived financial/logistic barriers	−0.013	−0.034
Total *R*^2^	0.174 ***	0.175 ***
Adjusted *R*^2^	0.140 ***	0.141 ***

Note. Numbers are standardized regression coefficients. Sex: male = 1, female = 2; Having heard of PM-related negative health effects: higher scores indicate greater levels of having heard of different negative health effects; Region: Seoul = 1, Pusan = 2, Daegu = 3, Incheon = 4, Gwangju = 5, Daejeon = 6, Ulsan = 7. Significance indicated by ^†^ *p* < 0.10, * *p* < 0.05, ** *p* < 0.01, *** *p* < 0.001.

## Data Availability

The data that support the findings of this study are available from the corresponding author, J.K., upon request.
